# Surface Vulnerability of Cerebral Cortex to Major Depressive Disorder

**DOI:** 10.1371/journal.pone.0120704

**Published:** 2015-03-20

**Authors:** Daihui Peng, Feng Shi, Gang Li, Drew Fralick, Ting Shen, Meihui Qiu, Jun Liu, Kaida Jiang, Dinggang Shen, Yiru Fang

**Affiliations:** 1 Division of Mood Disorders, Shanghai Mental Health Center, Shanghai Jiao Tong University School of Medicine, Shanghai, China; 2 Department of Radiology and BRIC, University of North Carolina, Chapel Hill, North Carolina, United States of America; 3 Suicide Research and Prevention Center, Shanghai Mental Health Center, Shanghai Jiao Tong University School of Medicine, Shanghai, China; 4 Palo Alto University, Palo Alto, California, United States of America; 5 The Fifth People’s Hospital of Shanghai, Fudan University, Shanghai, China; 6 Huashan Hospital, Fudan University, Shanghai, China; Beijing Normal University,Beijing, CHINA

## Abstract

Major depressive disorder (MDD) is accompanied by atypical brain structure. This study first presents the alterations in the cortical surface of patients with MDD using multidimensional structural patterns that reflect different neurodevelopment. Sixteen first-episode, untreated patients with MDD and 16 matched healthy controls underwent a magnetic resonance imaging (MRI) scan. The cortical maps of thickness, surface area, and gyrification were examined using the surface-based morphometry (SBM) approach. Increase of cortical thickness was observed in the right posterior cingulate region and the parietal cortex involving the bilateral inferior, left superior parietal and right paracentral regions, while decreased thickness was noted in the parietal cortex including bilateral pars opercularis and left precentral region, as well as the left rostral-middle frontal regions in patients with MDD. Likewise, increased or decreased surface area was found in five sub-regions of the cingulate gyrus, parietal and frontal cortices (e.g., bilateral inferior parietal and superior frontal regions). In addition, MDD patients exhibited a significant hypergyrification in the right precentral and supramarginal region. This integrated structural assessment of cortical surface suggests that MDD patients have cortical alterations of the frontal, parietal and cingulate regions, indicating a vulnerability to MDD during earlier neurodevelopmental process.

## Introduction

Major depressive disorder (MDD) is a disabling condition that includes symptoms of negative emotion and dysfunctional cognition. Major depressive disorder (MDD) is also a major public health concern, with a lifetime prevalence of about 16% [1]. Despite the widespread nature of this illness, clinical psychiatrists still face difficulty in the early identification of MDD. This is in part due to unclear underlying neurobiological mechanisms in MDD. In an effort to uncover these neurobiological mechanisms, researchers are increasingly utilizing neuroimaging methods, which indicate that regional alterations of brain structures are involved in depression [2].

One neuroimaging method is voxel-based morphometry (VBM). Previous studies using VBM reported that patients with MDD showed reduced grey matter volume in the prefrontal cortex [3], as well as reduced sub-cortical grey matter, (e.g., the thalamus and amygdala) [4]. However, other reports indicated that there was no change of grey matter volume in late-life depression [5, 6]. A possible reason of these discrepancies is that volume measurements from VBM are simultaneously contributed by many factors such as cortical thickness, surface area, and cortical folding, which have distinct genetic and cellular mechanisms [7, 8]. This highlights the importance of finding novel methods to study cortical morphologies in MDD specific samples.

Surface-based morphometry (SBM) is one such novel approach that enables researchers to capture multidimensional images of the brain in vivo. SBM can separately examine cortical thickness, surface area and cortical folding [8]. These three measurements provide specific indices that may reflect distinct developmental trajectories of cortex structures [9–11]. First, cortical thickness might be a sensitive measurement of neuronal density [12, 13], which has been used to assess mental disorders and related pathological changes [14, 15]. A majority of previous studies focused on late-onset depression, and found a decrease of cortical thickness in several regions, such as anterior cingulate cortex (ACC), posterior cingulate cortex (PCC), dorsolateral prefrontal cortex (DLPFC), and superior and middle temporal cortex [16–18]. However, these findings cannot be replicated by other studies even when using the same samples [5, 6]. Interestingly, these thinning cortical regions were partially observed in studies of non late-onset depression including first-episode [19–23] and/or recurrent samples [19–23]. Counter to these findings, one recent study found thicker middle frontal gyrus and ACC in depressive patients [19–23]. Second, the local surface area reflects the number and spacing of cortical columns in a cortex region [24]. Qiu et al. reported an increase in the surface area of the left parahippocampal gyrus in MDD patients [21]. However, there is currently a lack of other studies examining the surface area of patients with depression. Third, the local gyrification index (LGI) used to assess the degree of local cortical folding may be related to the developing integrity of cortical and subcortical circuits [25]. Previous studies of gyrification in depression provide various findings (e.g., decreased LGI in the bilateral mid-posterior cingulate, insular [26] and medial surface regions including the precuneus regions [27]) as well as increased LGI in the anterior regions (e.g., the left ACC [27]). To date, studies of cortical surface anatomical patterns show discrepant findings in MDD.

The dysfunction of the “cortical-limbic circuit” has been implicated in depressive emotion and accompanying cognitive deficits in depression [28–30]. The structural development of limbic and prefrontal regions was further confirmed to be associated with the onset of adolescent depression [31]. Cortical structures may be the primary components of this circuit by regulating emotional and cognitive processes (e.g., prefrontal cortex and cingulate cortex [32, 33]). Although some reports have summarized structural changes in MDD (e.g., frontal cortex [2], parietal cortex [34, 35], and cingulate cortex [16–18]) the distinct alterations of cortical structures need to be thoroughly studied in MDD. In the present study, we hypothesized that MDD might have atypical cortical patterns involving cortical thickness, surface area or cortical folding, potentially located in the prefrontal, parietal and cingulate regions. To test this hypothesis, in terms of the confounding factors (e.g., duration of disease and medication), we conducted surface-based morphometry (SBM) analysis of the whole cortex to examine these three specific indices in first-episode, unmedicated MDD patients by comparing them with healthy controls.

## Subjects and Methods

### Subjects

Medication-naïve patients with MDD were recruited from the outpatient clinics at Huashan Hospital and Shanghai Mental Health Centre, Shanghai, China. Healthy subjects matched with patients for age, sex, and education level were recruited through posters displayed in the two psychiatric clinics. This study was approved by the Institutional Review Board of Shanghai Mental Health Center. Written informed consent was obtained from all participants, following description of the study. All recruits underwent interviews and assessments independently by two psychiatrists, including the Structured Clinical Interview for DSM-IV (SCID), 24-item Hamilton Depression Scale (HAMD) [36, 37], and 14-item Hamilton anxiety scale (HAMA).

Inclusion criteria for depressed subjects were as follows: (a) aged 25–50 years; (b) met DSM-IV diagnosis criteria of MDD, first episode; (c) medication-naïve (d) 24-HAMD score > 20 [36], 14-HAMA score < 7, and (e) seeking outpatient treatment. Exclusion criteria were as follows: (a) currently or in the past meeting criteria for any other DSM-IV Axis I disorder (e.g., schizophrenia, schizoaffective disorder, bipolar disorder, or anxiety disorder as primary diagnosis); (b) taking any prescription or psychotropic medications in the past 4 weeks; (c) acutely suicidal, homicidal or requiring inpatient treatment; (d) meeting criteria for substance dependence within the past year (excluding caffeine or nicotine); (e) positive urinary toxicology screening at baseline; (f) use of alcohol in the past week; (g) serious medical or neurological illness; (h) currently pregnant or breastfeeding or (i) metallic implants or other contraindications to magnetic resonance imaging (MRI).

Healthy controls (HC) were included if they met the following criteria: (a) aged 25–50 years; (b) no history of psychiatric illness or substance abuse/dependence; (c) no family history of major psychiatric or neurological illness in first degree relatives; (d) not currently taking any prescription or psychotropic medications; (e) no use of alcohol in the past week and; (f) no serious medical or neurological illness. Exclusion criteria for the control group included: (a) pregnant or breastfeeding, or; (b) metallic implants or other contraindications that inhibited MRI.

In total 16 patients with MDD and 16 healthy controls were recruited using the above screening procedures, as well as the inclusion/exclusive criteria. All participants were verified as being right-handed with the right eye being dominant.

### Data acquisition

Participants were scanned using a 3.0-T General Electric Signa scanner with a standard whole-head coil. A high-resolution T1-weighted spoiled grass gradient sequence was used to acquire structural data. The imaging parameters were: 146 coronal slices, 1 mm thickness, slice gap = 0, repeat time (TR) = 500 ms, echo time (TE) = 14 ms, flip angle = 15°, field of view = 24×24 cm^2^, and image resolution = 0.9375×0.9375×1 mm^3^. The scanning time was 5 minutes.

### Data processing

The FreeSurfer software package was used to process the images (version 5.3.0, http://surfer.nmr.mgh.harvard.edu/). The procedures were based on previous reports [38]. Briefly, it included removal of non-brain voxels from each subject’s T1-weighted magnetic resonance imaging scan, transformation to standard space, and segmentation of the grey-white matter tissues. The grey-white boundary was represented with a polygonal tessellation, and topological correction was performed. This surface was then deformed outwards to locate the pial surface (i.e., boundary between cerebrospinal fluid (CSF) and grey matter). We reviewed all obtained cortical surfaces, and minimal manual editing was performed at inaccurate segmentations. The generated cortical surfaces were validated by comparing them to the manual measures on MRI data [38–40]. Cortical thickness was computed at each vertex as the average distance between the grey-white surface and pial surface. Surface area for each vertex was calculated on the pial surface, representing the area of the tessellated triangles linked to the vertex. The local cortical folding for each vertex was measured by means of local gyrification index (LGI), which took into account the ratio of local surface area to the outer hull layer that tightly wraps the pial surface [41], and was extended from the two-dimensional gyrification measurement [42]. The LGI value is an indication of sulcal cortex buried in its locality and thus denotes the extent of cortical folding [40].

### Statistical analysis

SPSS 17.0 was used for statistical analysis. Two sample *t*-tests were used to compare the demographic characteristics between groups. Chi-square test was used to measure the difference of gender distribution. For each vertex, a general linear model (GLM) was used to detect the significance of differences in cortical thickness, surface area, and LGI between the patients with MDD and controls, respectively. The confounding factors were regressed out, including age, gender, education, and an overall measurement. This overall measurement refers to the mean cortical thickness, total surface area, and average LGI of each individual subject, respectively. The smoothing kernel of 15 mm was applied before group comparisons at the level of each vertex.

## Results

The demographic and clinical data are summarized in Table 1. The two groups were matched for age, gender, and education level (*p*>0.05). Mean age of onset was 33.25 ± 6.98, and the mean duration of illness was 8.01 ± 4.19 weeks in the MDD group. The MDD group had significantly higher HAMD scores and higher HAMA scores than the control group (*p*<0.0001).

**Table 1:** Demographic and clinical characteristics.

	Depressed patients (n = 16)	Healthy subjects (n = 16)	Significance (*P*)
Age (years)	34.43 ± 6.72	33.75 ± 6.36	0.77
Gender (male/female)	7/9	7/9	—
Education level	15.63±1.99	15.81±2.48	0.82
Age on set (years)	33.25 ± 6.98	—	—
Duration of illness (weeks)	8.01± 4.19	—	—
24-item HRSD Score	30.88 ± 7.69	3.81±1.05	<0.0001*
14-item HAMA Score	5.62±0.72	3.31±1.25	<0.0001*

Significance was evaluated using two sample *t*-test.

* denotes significance found.

### Cortical thickness

**Fig 1:**
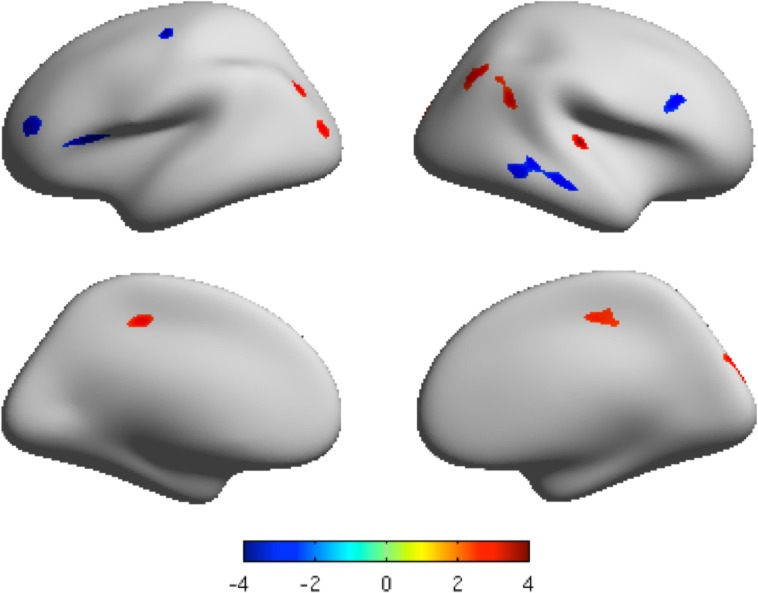
The clusters with significantly different cortical thickness projected onto the average cortical surface. Lateral view and medial view by inflated surfaces, color-coded by t-scores. Red means that MDD has higher thickness than that of controls, and blue means that MDD has lower thickness than that of controls. *P* < 0.05, FDR corrected, cluster size > 200 vertices.

The mean cortical thickness of both the MDD group and healthy control group is shown in Fig A in S1 File. Both significantly increased and decreased cortical thickness was found in MDD patients compared to healthy controls (*p*<0.05 by multiple comparisons correction using False Discovery Rate (FDR), cluster size>200 vertices) [43]. These cortices with increased thickness included the right inferior parietal region, right paracentral region, right transverse temporal region, right posterior cingulate region, left superior parietal region, left inferior parietal region, and left lateral occipital region. Those with decreased thickness were located in the right middle temporal region, right pars opercularis, left pars opercularis region, left rostral-middle frontal region, and left precentral region (Table 2, Fig. 1 and Fig. D in S1 File).

**Table 2:** Cortex areas with altered cortical thickness in patients with major depressive disorder, compared to healthy controls.

Regions	Hemisphere	Cluster size	MNI coordinates (Maximum vertex)			*t* Value
			x	y	z	
**MDD>HC**						
Inferior parietal	R	698	42	−67	48	3.28
	R	390	50	−52	24	3.14
Paracentral	R	557	14	−24	42	2.89
Superior parietal	L	473	13	−92	23	2.97
Transverse temporal	R	318	50	−22	7	3.93
Inferior parietal	L	295	−31	−82	33	3.15
Posterior cingulate	R	216	1	−3	35	2.80
Lateral occipital	L	212	−34	−86	6	3.14
**HC> MDD**						
Middle temporal	R	947	68	−37	−5	−2.47
Pars percularis	L	559	−43	15	1	−2.47
Rostral−middle frontal	L	353	−40	47	20	−2.47
Precentral	L	306	−38	−15	51	−2.48
Pars opercularis	R	283	42	19	21	−2.47

MDD: major depressive disorder. HC: healthy control. L: left; R: right. MNI: Montreal Neurological Institute. *P* < 0.05 FDR corrected, Cluster size > 200 vertices.

### Surface area

**Fig 2:**
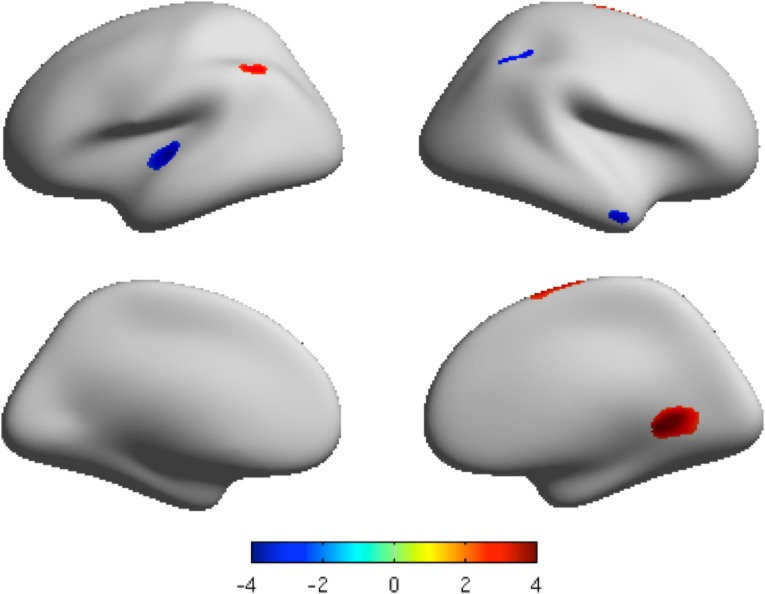
The clusters with significant different local surface area projected onto the average cortical surface. Lateral view and medial view by inflated surfaces, color-coded by t-scores. Red means that MDD has higher surface area than that of controls, and blue means that MDD has lower surface area than that of controls. *P* < 0.05 FDR corrected, cluster size > 200 vertices.

The mean surface area of both the MDD group and healthy control group is shown in Fig. B in S1 File. The cortices with significantly increased surface area in patients with MDD (as compared with healthy controls) included the right isthmus cingulate region, right superior frontal region, and left inferior parietal region. In contrast, the cortices with significantly decreased surface area in patients with MDD included the left transverse temporal region and right inferior parietal region. The statistical threshold was set to *p* < 0.05 with FDR corrected and cluster size > 200 vertices (Table 3, Fig. 2, Fig. E in S1 File).

**Table 3:** Cortex areas with altered cortical surface areas in patients with major depressive disorder, compared to healthy controls.

Region	Side	Cluster size	MNI coordinates (Maximum vertex)			*t* Value
			x	y	z	
**MDD>HC**						
Isthmus cingulate	R	1473	16	−48	−1	3.80
Superior frontal	R	958	15	−4	72	2.94
Inferior parietal	L	799	−50	−59	44	3.17
**HC> MDD**						
Transverse temporal	L	754	−40	−24	1	−2.47
Inferior parietal	R	359	38	−48	40	−2.47

MDD: major depressive disorder. HC: healthy control. L: left; R: right. MNI: Montreal Neurological Institute. *P* < 0.05 FDR corrected, Cluster size > 200 vertices.

### Local gyrification index

**Fig 3:**
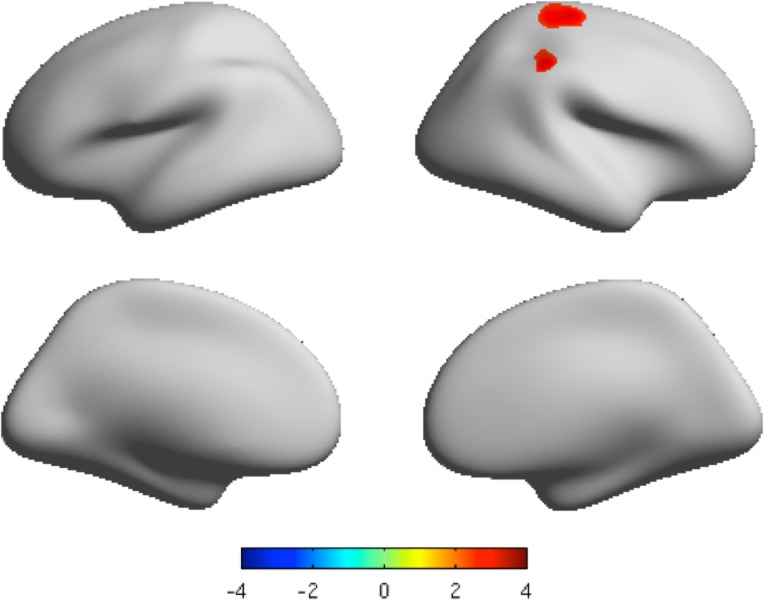
The clusters with significant different LGI projected onto the average cortical surface. Lateral view and medial view by inflated surfaces, color-coded by t-scores. Red means that MDD has higher local gyrification than that of controls. *P* < 0.05 FDR corrected, cluster size > 200 vertices.

The mean LGI of both the MDD group and healthy control group is shown in Fig. C in S1 File. There was significant hyper-gyrification in the MDD group in the right precentral and supramarginal regions in comparison to the healthy group (*P* < 0.05, FDR corrected, and cluster size > 200 vertices) (Table 4, Fig. 3, Fig. F in S1 File). No lower level of gyrification was found in MDD group.

**Table 4:** Brain areas with different local gyrification index in patients with major depressive disorder, compared to healthy controls.

Region	Side	Cluster size	MNI coordinates (Maximum vertex)			*t* Value
			x	y	z	
**MDD>HC**						
Precentral	R	2011	24	−27	53	3.17
Supramarginal	R	564	50	−37	49	2.81

MDD: major depressive disorder. HC: healthy control. L: left; R: right. MNI: Montreal Neurological Institute. *P* < 0.05 FDR corrected, Cluster size > 200 vertices.

## Discussion

To the best of our knowledge, this is the first multidimensional assessment of cortical anatomy in first-episode, MDD patients without medication, measuring cortical thickness, surface area and cortical folding. Although these three cortical attributes reflect different aspects of neurobiological developments of cortical structures, atypical sub-regions, located in the frontal, parietal, and cingulate cortexes, were commonly observed in MDD. These regions have been assumed to regulate function of the cortical-limbic circuit, which may contribute to clinical symptoms of depression (e.g., depressive emotion and dysfunctional cognitions) [28–30]. These disrupted alterations of cortical structures are helpful to delineate the neuropathological process of MDD during the early stage.

### Cortical thickness

We observed seven regions with increased cortical thickness. The current findings are partly consistent with two recent studies of first-episode MDD showing increased cortical thickness in the temporal pole, and cingulate cortex [44], as well as in the frontoparietal regions, and supramarginal gyrus [21]. First, increased thickness of the parietal cortex included four sub-regions: bilateral inferior parietal, left superior parietal, and the right paracentral region, which is believed to play a role in the interpretation of sensory information (e.g. the perception of emotions in facial stimuli) [45]. MDD patients usually suffer from atypical emotional perception, which may be related to changes in the parietal lobe [46, 47]. Second, the transverse temporal cortex is linked to the primary sensory cortical areas and processes auditory information [48, 49]. Norepinephrine, as one of the neurotransmitters that modulate the function of the primary auditory cortex [48], has been shown to be decreased in patients with MDD [50, 51]. Third, the lateral occipital cortex is involved in the processing of visual information. MDD is often characterized by negative response to emotional stimuli in the visual cortex [52]. Finally, the posterior cingulate region may hold an important role among all these regions with increased cortical thickness. The posterior cingulate region is a core region of the default mode network (DMN) constructed by resting-state fMRI data [53]. The DMN integrates aforementioned regions, such as the parietal cortex and the joint regions of both temporal and occipital cortices. This functional network is responsible for self-directed mental processes [54, 55]. Converging evidence has indicated abnormal DMN in patients with MDD [56–58]. Since structural connectivity has been shown to be the base of a functional network [59], our findings provide structural level evidence that supports a connection between atypical DMN and the mental process seen in MDD, such as abnormal emotional perception of visual-auditory stimuli.

The decrease of cortical thickness was observed in two areas located in the right hemisphere (middle temporal, and pars opercularis regions) and three areas in the left hemisphere (involving the rostral-middle frontal, precentral and pars opercularis regions). A recent study did not observe any decreased cortical thickness in patients with untreated first-episode MDD [21]. This discrepancy might be attributed to the sample heterogeneity between these two studies. The mean duration of illness in our cases was 8 weeks, which is significantly shorter than the mean duration of illness of 17.4 weeks seen in Qiu et al’s study [21]. Despite the above mentioned differences, our findings of decreased cortical thickness are supported by multiple studies of late-onset depression [16–18]. Our study found decreased cortical thickness in the following areas. First, the rostral-middle frontal region (which is related to the DLPFC) is involved in the cognitive processes of attention and memory [60]. Second, the bilateral pars opercularis form a portion of Broca’s area together with the frontal operculum that is responsible for the processing of speech-language [61]. Third, the middle temporal region is part of extra-striate visual cortex, which is known to be connected with the recognition of familiar faces [62, 63]. Finally, the precentral region plays a role in the planning and executing of movements, and is associated with other motor areas (e.g., supplementary motor and premotor cortex) [64]. Interestingly, deficits of cognitive processing (e.g., attention and working memory) are often observed in patients with MDD [65, 66]. Patients with MDD often also suffer from symptoms of psychomotor retardation [67–69]. These symptoms of dysfunctional cognition and psychomotor retardation may be observed in both first-episode and late-onset depression; the decreased cortical thickness found in our first-episode cases is also located in most of those regions with thinner cortex reported in the late-onset depression cortex [16–18]. This evidence could indicate that anatomical alterations predict the structural markers in MDD, and are further linked to clinical symptoms (e.g., cognitive defects and retardation) seen in MDD. However, these two issues remain to be determined in longitudinal studies.

### Cortical Surface Area

There are few reports on the cortical surface area in MDD that can be used to compare with the present findings. An aforementioned study revealed an increasing trend of surface area in the left parahippocampal gyrus in MDD [21], which was not replicated in our finding. During different periods of illness, there may be different spatial patterns of the cortical surface area. The significantly earlier stage of illness of our cases may explain the inconsistency between these two studies. In our study, increased surface area was observed in the right isthmus cingulate cortex and superior frontal region, as well as in the left inferior parietal cortex. In contrast, decreased surface area was found in the left transverse temporal region and the right inferior parietal region. There are several regions overlapping with each other among those cortical areas with altered surface area or cortical thickness (e.g., the frontal region, parietal region and cingulate cortex) while the regions with altered surface area are partially inconsistent with the regions with altered cortical thickness. These two anatomical measurements reflect different patterns of the structural physiology, but in patients with MDD structural alterations were found in similar specific regions in the cortex when using a cortical thickness index or a surface area index. Furthermore, the cortical volumes can be calculated by combining these two parameters. Most of the previous studies on MDD reported reduced cortical volumes, in areas such as the prefrontal cortex [3], thalamus and amygdala [4]. Several studies of MDD also reported increased volumes in certain areas (e.g., in the precuneus and angular gyrus) [70, 71]. Differences of sample characteristics, such as medication effects, level of severity and duration of illness, might impact the morphometric patterns. Although the measures are different between cortical volume and surface patterns, the consistent findings among different studies suggests that surface indices contribute to variance in cortical volume. Previous reports of altered cortical volume in MDD predicted the possibility of both increased and decreased surface areas among different cortical regions. Thus, our findings of surface area may provide one potential reference in cortical changes for early stage MDD.

### Local Gyrification Index

This study observed increased LGI in the MDD group located in the right precentral region and supramarginal region, which were also regions overlapping the areas with altered cortical thickness. Our findings were not consistent with two previous studies of LGI in depression. In patients with recovered-state MDD, Nixon *et al* found decreased LGI in the precuneus and increased LGI in the left ACC [27]. Among first-episode MDD samples without medication, Zhang *et al* reported that the MDD group exhibited decreased LGI in specific regions (e.g., the frontal cortex and the cingulate regions) [26]. The illness durations of samples are different among these three studies. The Nixon’s report was to explore the cortical gyrification abnormalities within recovered-state MDD [27]. Both our samples and the samples in Zhang’s study are first episode. However, our samples are during the acute period (2.0±1.1 months), unlike the participants in Zhang’s study (6.7±3.9 months) [26]. Importantly, the LGI used to assess the degree of local cortical folding may be related to the developing integrity of cortical and subcortical circuits [25]. As during different duration of illness, the patterns of integrity might be different, the differences in results could reflect the different integrity related to illness duration. The cortical folding results from the differential growth rates among all kinds of cortical layers during brain convolution development [72]. The axon tension in white matter also affects the folding patterns during cortex development [73]. Theoretically, the defects of gyrification may reflect atypical neurodevelopment in MDD. Thus, the hypergyrification found in the right precentral and supramarginal regions in this study likely represents the earlier alterations of cortical folding in MDD.

The precentral gyrus with hypergyrification observed in this study is part of the primary motor cortex, which is relevant to the initiation of movement by integrating information from the sensorimotor cortex [74]. The large corticospinal neurons typically present in this region [75], while the dopaminergic projection to the primary motor cortex mediates the biochemical pathway of movement [76]. Interestingly, psychomotor retardation is often observed in the MDD described above as an important clinical symptom because of dysfunctional dopamine pathways [77–79]. In a diffusion tensor imaging study, an association was confirmed between psychomotor retardation and alterations of white matter pathways involving the motor cortex [68]. Using fMRI methods, patients with MDD showed increased functional connectivity among the precentral gyrus and other sensorimotor systems (e.g., DLPFC and ACC) [80]. Although the methodologies are different, the findings of these neuroimaging studies may be helpful in explaining the clinical meanings of hypergyrification within this region in MDD. This finding further supports a notion that depression is characterized by atypical function of the sensory-motor circuit, using the evidence of altered gyrification in the precentral region [81].

A recent study extends the basic function of the supramarginal area to the social cognition processes related to affective states [82]. There are fewer reports on altered supramarginal gyrus in MDD, although one recent study of MDD found thicker cortex in the right supramarginal region [21]. Likewise, humans use the self as a reference to perceive the world based on the self-referential projection model [83, 84]. Clinically, patients with MDD often are characterized by disrupted self-rumination [85, 86], and over-general autobiographical memory (OGM) from negative life events [87]. The hypergyrification in the supramarginal region found in this study may demonstrate a novel explanation for the aforementioned social cognitive symptoms accompanying MDD.

Although this study provides somewhat new insight into the cortical surface patterns in MDD, there are still some limitations in our study. First, the current findings contain many regions of the cerebral cortex. We need to further focus on the specific brain areas with potential biomarkers by combining other approaches (e.g., neurotransmitter and neuropathology). Second, sub-cortical structures, such as the thalamus and amygdala, that are supposed to underline some clinical characteristics of MDD, cannot be measured using this approach. Third, our sample size is small and therefore any structural changes found in the cortex should be interpreted with caution; we plan to recruit more samples with MDD to further investigate this issue. Finally, our current study is cross-sectional in design. As the morphological patterns may be varied in different stages of illness, further longitudinal studies are needed to differentiate the variations underlying the illness periods.

## Conclusions

The present study extends previous anatomical investigations of MDD by using multidimensional assessments of the whole cortex. Our findings identify specific cortical areas with surface morphological alterations in first-episode MDD patients not taking medication, mainly involving the frontal region, parietal region, and cingulate region. The atypical surface patterns confirm our hypothesis that the cortical changes may be observed by multiple parameters of cortical thickness, surface area, and cortical folding, and may predict the earlier neurodevelopmental markers in MDD. The underlying pathophysiological connections of these alterations in the cortical surface to clinical symptoms (e.g., negative emotional process, dysfunctional cognition, and psychomotor retardation) need to be further clarified.

## Supporting Information

S1 File Supporting Information file containing Figures A-F. **Fig. A**. Average cortical thickness (mm) on the whole brain cortices of both MDD patient group and healthy control group. MDD: major depressive disorder; HC: healthy control. **Fig. B**. Average local surface area (mm^2^) on the whole brain cortices of both MDD patient group and healthy control group. MDD: major depressive disorder; HC: healthy control. **Fig. C**. Average local gyrification index on the whole brain cortices of both MDD patient group and healthy control group. MDD: major depressive disorder; HC: healthy control. **Fig. D**. The clusters with significantly different cortical thickness projected onto the average cortical surface. Lateral view and medial view by original surfaces, color-coded by t-scores. Red means that MDD has higher thickness than that of controls, and blue means that MDD has lower thickness than that of controls. *P* < 0.05, FDR corrected, cluster size > 200 vertices. **Fig. E**. The clusters with significant different local surface area projected onto the average cortical surface. Lateral view and medial view by original surfaces, color-coded by t-scores. Red means that MDD has higher surface area than that of controls, and blue means that MDD has lower surface area than that of controls. *P* < 0.05 FDR corrected, cluster size > 200 vertices. **Fig. F**. The clusters with significant different LGI projected onto the average cortical surface. Lateral view and medial view by original surfaces, color-coded by t-scores. Red means that MDD has higher local gyrification than that of controls. *P* < 0.05 FDR corrected, cluster size > 200 vertices.
(DOC)
