# Correction: Surface Vulnerability of Cerebral Cortex to Major Depressive Disorder

**DOI:** 10.1371/journal.pone.0128947

**Published:** 2015-06-29

**Authors:** Daihui Peng, Feng Shi, Gang Li, Drew Fralick, Ting Shen, Meihui Qiu, Jun Liu, Kaida Jiang, Dinggang Shen, Yiru Fang

There are errors in the author affiliations. The affiliations should appear as shown here:

Daihui Peng^1,2^, Feng Shi^2^, Gang Li^2^, Drew Fralick^3,4^, Ting Shen^1^, Meihui Qiu^1^, Jun Liu^5^, Kaida Jiang^6^, Dinggang Shen^2,7^, Yiru Fang^1^

1 Division of Mood Disorders, Shanghai Mental Health Center, Shanghai Jiao Tong University School of Medicine, Shanghai, China, 2 Department of Radiology and BRIC, University of North Carolina, Chapel Hill, North Carolina, United States of America, 3 Suicide Research and Prevention Center, Shanghai Mental Health Center, Shanghai Jiao Tong University School of Medicine, Shanghai, China, 4 Palo Alto University, Palo Alto, California, United States of America, 5 The Fifth People’s Hospital of Shanghai, Fudan University, Shanghai, China, 6 Huashan Hospital, Fudan University, Shanghai, China 7 Department of Brain and Cognitive Engineering, Korea University, Seoul, Republic of Korea
