# Supplementary material for: Surface Vulnerability of Cerebral Cortex to Major Depressive Disorder
Source: PLoS One. 2015 Mar 20;10(3):e0120704. doi: 10.1371/journal.pone.0120704 (PMC4368815; doi:10.1371/journal.pone.0120704)
Supplement: S1 File — Fig. A. Average cortical thickness (mm) on the whole brain cortices of both MDD patient group and healthy control group. MDD: major depressive disorder; HC: healthy control. Fig. B. Average local surface area (mm2) on the whole brain cortices of both MDD patient group and healthy control group. MDD: major depressive disorder; HC: healthy control. Fig. C. Average local gyrification index on the whole brain cortices of both MDD patient group and healthy control group. MDD: major depressive disorder; HC: healthy control. Fig. D. The clusters with significantly different cortical thickness projected onto the average cortical surface. Lateral view and medial view by original surfaces, color-coded by t-scores. Red means that MDD has higher thickness than that of controls, and blue means that MDD has lower thickness than that of controls. P < 0.05, FDR corrected, cluster size > 200 vertices. Fig. E. The clusters with significant different local surface area projected onto the average cortical surface. Lateral view and medial view by original surfaces, color-coded by t-scores. Red means that MDD has higher surface area than that of controls, and blue means that MDD has lower surface area than that of controls. P < 0.05 FDR corrected, cluster size > 200 vertices. Fig. F. The clusters with significant different LGI projected onto the average cortical surface. Lateral view and medial view by original surfaces, color-coded by t-scores. Red means that MDD has higher local gyrification than that of controls. P < 0.05 FDR corrected, cluster size > 200 vertices. (DOC) [file pone.0120704.s001.doc]

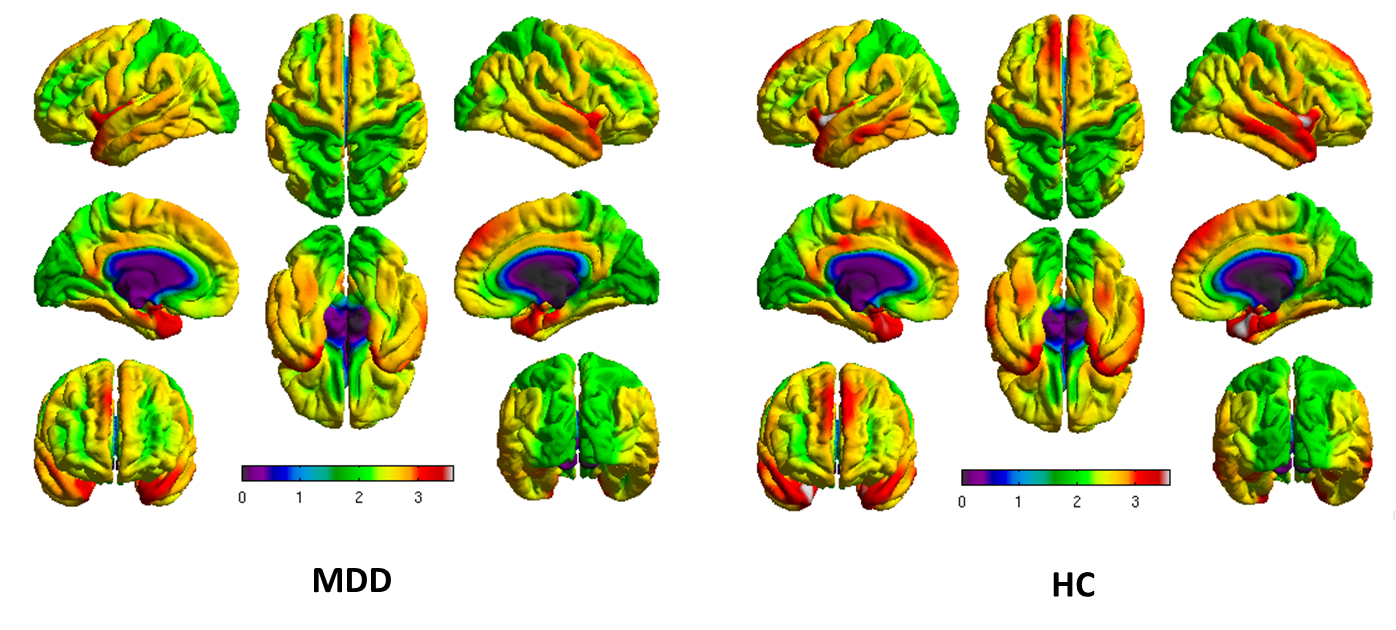


**Fig. A. Average cortical thickness (mm) on the whole brain cortices of both MDD patient group and healthy control group.** MDD: major depressive disorder; HC: healthy control.


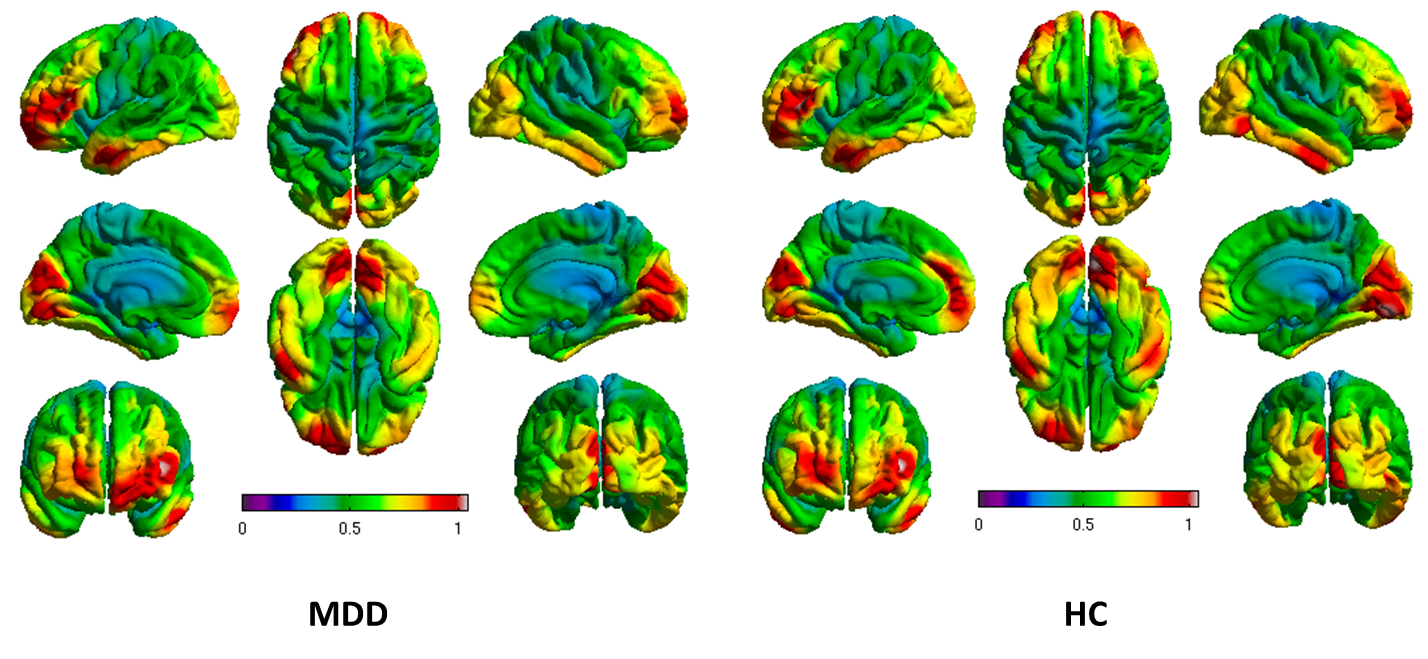


**Fig.B. Average local surface area (mm^2^) on the whole brain cortices of both MDD patient group and healthy control group.** MDD: major depressive disorder; HC: healthy control.


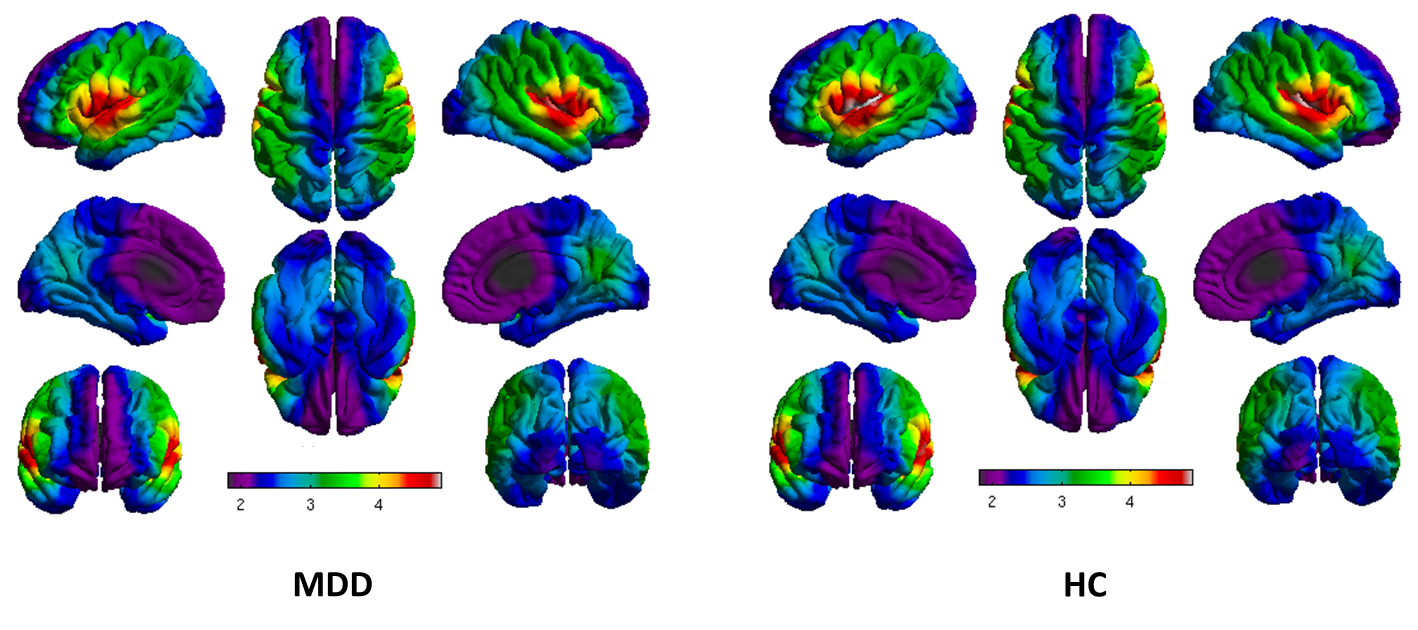


**Fig.C. Average local gyrification index on the whole brain cortices of both MDD patient group and healthy control group.** MDD: major depressive disorder; HC: healthy control.


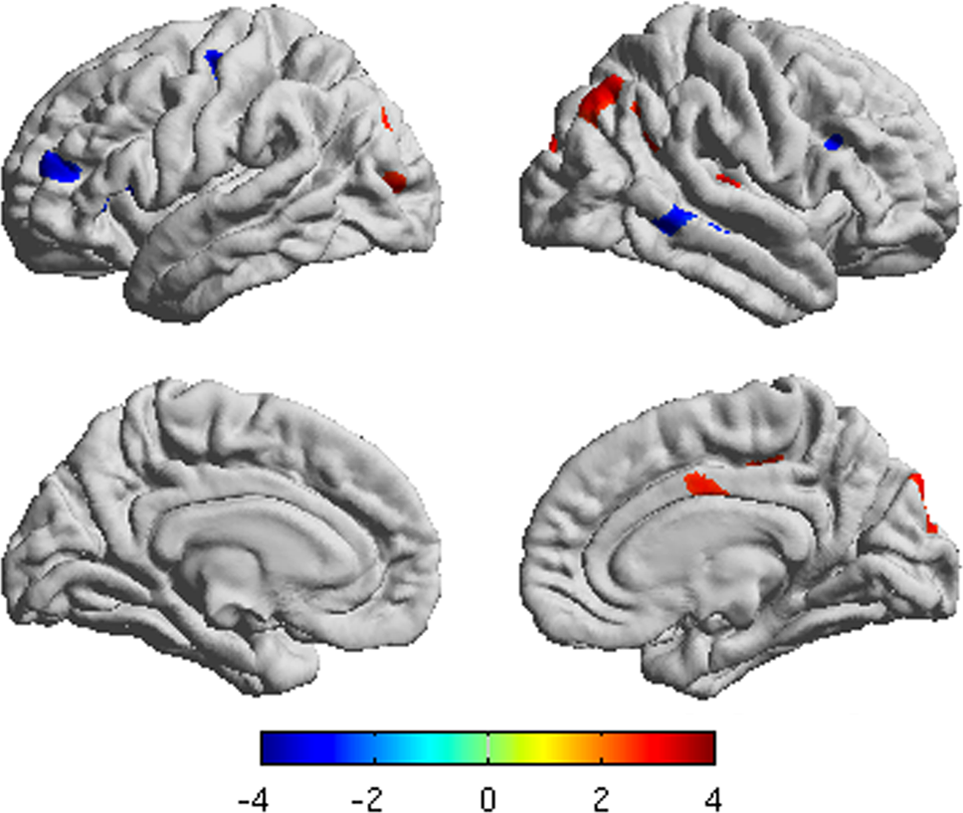


**Fig.D. The clusters with significantly different cortical thickness projected onto the average cortical surface.** Lateral view and medial view by original surfaces, color-coded by t-scores. Red means that MDD has higher thickness than that of controls, and blue means that MDD has lower thickness than that of controls. *P* < 0.05, FDR corrected, cluster size > 200 vertices.


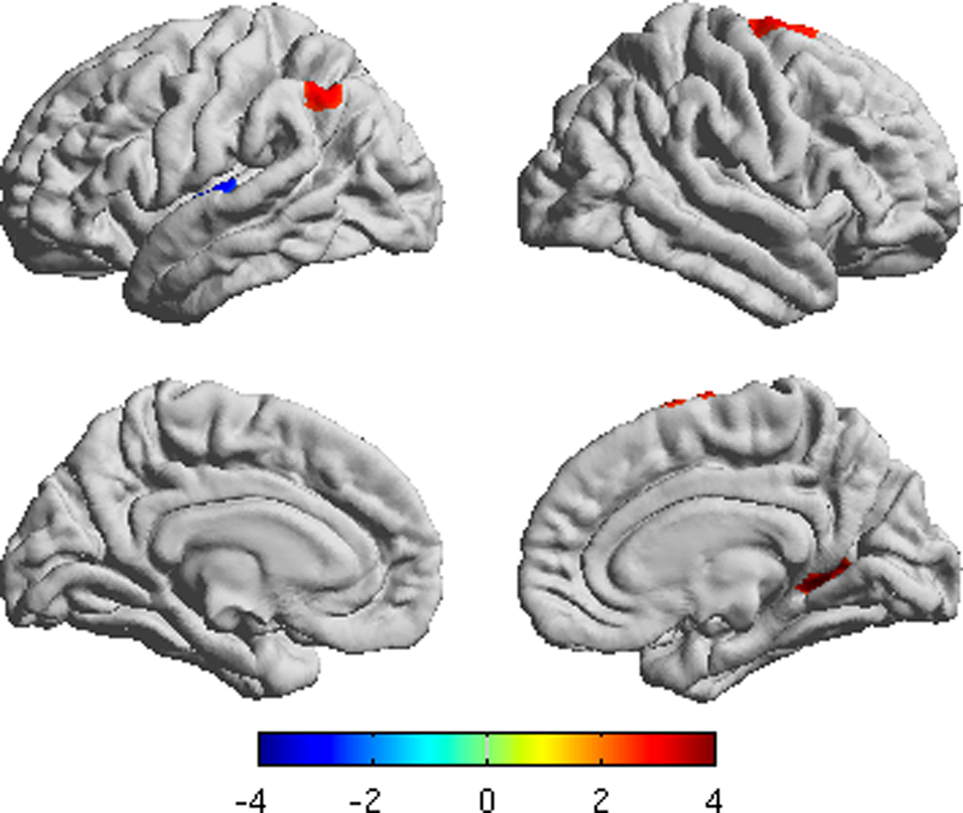


**Fig.E. The clusters with significant different local surface area projected onto the average cortical surface.** Lateral view and medial view by original surfaces, color-coded by t-scores. Red means that MDD has higher surface area than that of controls, and blue means that MDD has lower surface area than that of controls. *P* < 0.05 FDR corrected, cluster size > 200 vertices.


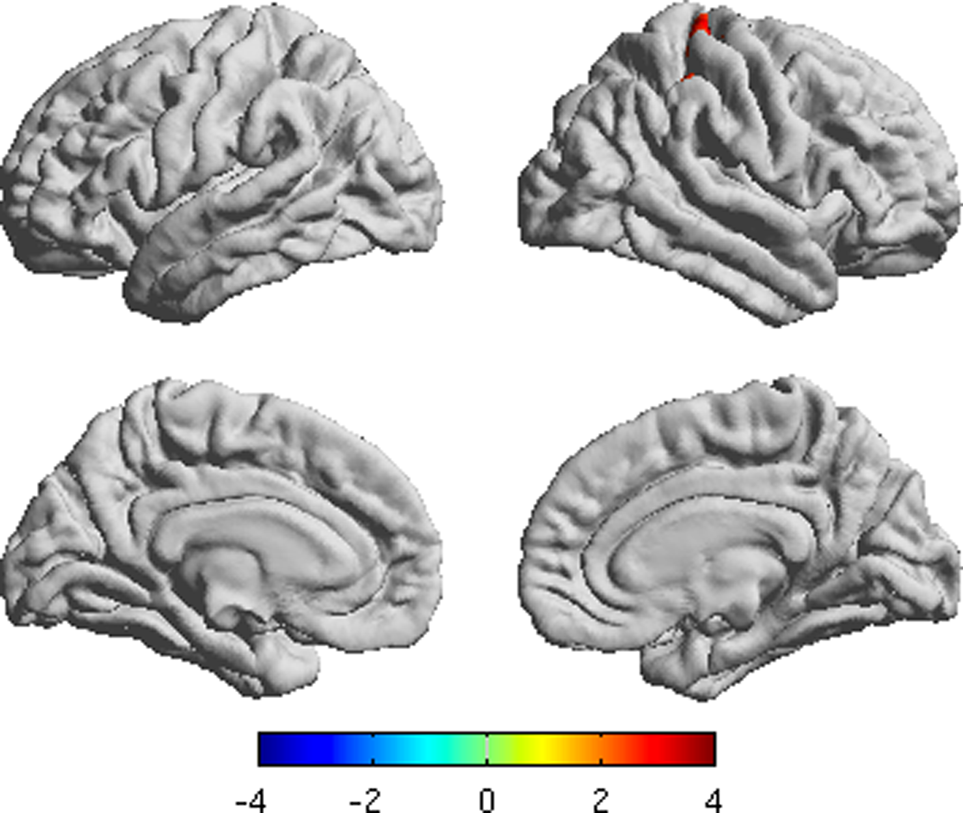


**Fig.F. The clusters with significant different LGI projected onto the average cortical surface. Lateral view and medial view by original surfaces, color-coded by t-scores.** Red means that MDD has higher local gyrification than that of controls. *P* < 0.05 FDR corrected, cluster size > 200 vertices.
